# Functional Analysis of *CsWOX4* Gene Mutation Leading to Maple Leaf Type in Cucumber (*Cucumis sativus* L.)

**DOI:** 10.3390/ijms252212189

**Published:** 2024-11-13

**Authors:** Huizhe Wang, Bo Wang, Yiheng Wang, Qiang Deng, Guoqing Lu, Mingming Cao, Wancong Yu, Haiyan Zhao, Mingjie Lyu, Ruihuan Yang

**Affiliations:** State Key Laboratory of Vegetable Biobreeding, Tianjin Academy of Agricultural Sciences, Tianjin 300192, China; wanghuizhe@126.com (H.W.); wangbo0426mm@163.com (B.W.); sywyhyx@126.com (Y.W.); dengqiang022@126.com (Q.D.); luguoqing007@163.com (G.L.); caoming2013@126.com (M.C.); yuwancong@sohu.com (W.Y.); changgjce@163.com (H.Z.)

**Keywords:** leaf morphology, *Cucumis sativus* L., map cloning, *CsWOX4* gene, BSA

## Abstract

The leaf morphology is an important agronomic trait in crop production. Our study identified a maple leaf type (*mlt*) cucumber mutant and located the regulatory gene for leaf shape changes through BSA results. Hybrid F1 and F2 populations were generated by F1 self-crossing, and the candidate *mlt* genes were identified within the 2.8 Mb region of chromosome 2 using map cloning. Through the sequencing and expression analysis of genes within the bulk segregant analysis (BSA) region, we identified the target gene for leaf shape regulation as *CsWOX4* (CsaV3_2G026510). The change from base C to T in the original sequence led to frameshift mutations and the premature termination of translation, resulting in shortened encoded proteins and conserved WUSCHEL (*WUS*) box sequence loss. The specific expression analysis of the *CsWOX4*/*Cswox4* genes in the roots, stems, leaves and other tissue types of wild-type (WT) and mutant plants revealed that *CsWOX4* was higher in the root, but *Cswox4* (mutant gene) was significantly higher in the leaf. Subcellular localization analysis revealed that CsWOX4 was localized in the nucleus. RNA-seq analysis revealed that the differentially expressed genes were mainly enriched in the mitochondrial cell cycle phase transition, nucleosome and microtubule binding pathways. Simultaneously, the quantitative analysis of the expression trends of 25 typical genes regulating the leaf types revealed the significant upregulation of *CsPIN3*. In our study, we found that the conserved domain of CsWOX4 was missing in the mutant, and the transcriptome data revealed that the expression of some genes, such as *CsPIN3,* changed simultaneously, thereby jointly regulating changes in the cucumber leaf type.

## 1. Introduction

China accounts for 81% of the global total production of cucumber and has the largest cucumber cultivation area and highest total yield. Cucumber is the most extensively cultivated vegetable crop in protected areas in China and thus holds an extremely important position in vegetable production [1]. The morphology of cucumber leaves is of great significance in understanding the growth and development of cucumbers and the close planting of cucumber plants. The leaves are the main organs for photosynthesis in plants, and increasing the leaf area enhances the efficiency of light absorption and utilization. The leaves also play a crucial role in various processes, including respiration [2]. Leaf morphogenesis is an important process in plant morphogenesis. The leaf morphology includes two important factors—the size and shape—which are crucial for crop formation and yield [3]. The leaf morphology is directly associated with the light utilization efficiency and affects the crop quality and yield [4]. The leaf shape has been a subject of concern in the field of breeding, and improvements in the plant leaf shape can achieve high-yield crop variety breeding. Therefore, the study of leaf morphogenesis contributes to a deeper understanding of the morphological mechanisms of plants’ lateral organs and provides a theoretical basis for techniques such as close planting.

The WUSCHEL-related homeobox (WOX) is a family of plant-specific transcription factors that contain a homeodomain (HD) consisting of 65–66 amino acid residues [5]. Previous searches for *WUS* homologous sequences in *Arabidopsis* databases revealed 14 genes *WOX1*–*WOX14* that encode the protein, which has a sequence similar to the WUS protein sequences [6]. WUS was the first gene discovered in the WOX family and is crucial in maintaining the integrity of the stem and inflorescence meristem structure and function in *Arabidopsis thaliana* [7,8].

WOX regulates plant leaf development, with WOX5, WOX1 and WOX3 in the *Arabidopsis* WOX family exhibiting redundant functions in the regulation of the leaf shape [9]. These WOX genes control the expression of YUCCA (*YUC*) auxin biosynthesis genes along the leaf edge region to form an off-axis growth gradient, thereby promoting lateral growth and resulting in the characteristic elliptical leaf shape of *Arabidopsis* [10,11]. A loss-of-function mutant of the rice WOX3 homologous gene leaf lateral symmetry 1 (*LSY1*) exhibited asymmetric defects during early leaf development, whereas *LSY1* overexpression resulted in deformed leaves with curled edges in rice [12]. The downregulation of OsWOX4 leads to serious defects in leaf development, such as arrested vascular differentiation, partial defects in early cell proliferation required for midrib formation and an inability to maintain cell activity in thin-walled cells [13]. Notably, the un-fused flower (uf) plant mutant of the *SlWOX1* gene in tomato resulted in the significant inhibition of the lateral growth of leaves, the curling of leaf edges towards the near axis and a significant increase in the number of small leaves per leaf [14].

Researchers have cloned and identified members of the WOX family in most plants (e.g., *Arabidopsis*, rice, alfalfa and poplar) and analyzed the biological functions of some of them [15]. Numerous studies have reported the involvement of WOX in the regulation of different structures in plants and various abiotic stresses [16]. However, research on the involvement of WOX family genes in leaf type regulation in cucumber remains limited, and related studies have mainly focused on plants such as *Arabidopsis*, rice, cotton and other field crops, such as rapeseed, potatoes and soybeans. Therefore, we discovered a natural leaf type mutant of cucumber in our previous work and generated an F2 population for BSA to identify candidate regulatory genes in cucumber. We also preliminarily predicted the regulatory mechanism of mutant leaf type formation. Our findings can be applied to the genetic improvement of important crop traits, which could facilitate screening for excellent genetic resources.

## 2. Results

### 2.1. Morphological Characteristics of K39

**Figure 1:**
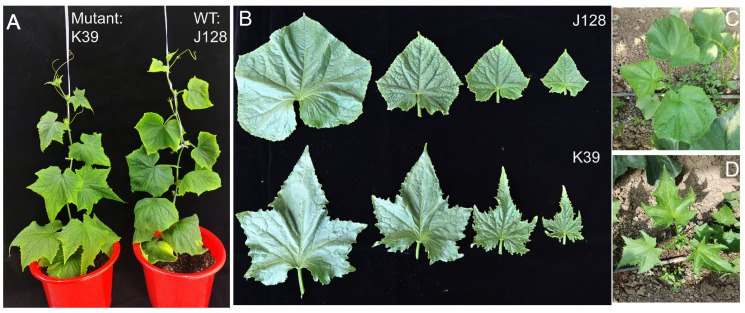
Mature and seedling stage characteristics of wild-type “J128” and K39 mutants. (**A**) Phenotypes of wild type and mutant during the seedling stage. (**B**), Different phenotypes of wild-type and mutant leaves. Top right: Leaf phenotype of ”J128” wild-type seedlings in the field. Lower right: Leaf phenotype of “K39” seedlings in the field. (**C**) J128 field growing leaf morphology. (**D**) K39 field growing leaf morphology.

The leaf type mutant K39 was derived from natural mutants obtained from the field. At the young leaf stage, the leaf type mutant underwent significant changes, with the leaves showing a concave inward shape. The leaves exhibited a concave shape during the period from young to mature leaves, which was very different from that of the WT leaves, and the leaf area was only approximately 50% of that of the round WT leaves (Figure 1A). Compared with the WT leaves of J128, the mutant had wrinkled and uneven leaves from the cotyledon stage, with wedge-shaped first and second true leaves. Starting from the third true leaf, the main leaf veins extended outward, and the leaf fissures deepened, resembling ginkgo leaves (Figure 1B). Subsequently, K39 was crossed with the WT J128 (male parent) to further study the genetic characteristics of the mutants. Notably, the F1 generation was fertile, whereas, in the F2 population, 66 plants with a mutant-type phenotype and 191 WT plants were obtained in a 1:2.894 ratio. The chi-squared (χ^2^) test revealed that the segregation ratios of the F2 population were consistent with the 3:1 Mendelian ratio. These results indicated that the mutant was controlled by a single nuclear recessive gene (Table 1).

### 2.2. Identification of mlt Using BSA Combined with Parent Transcriptome Sequencing Analysis

**Figure 2:**
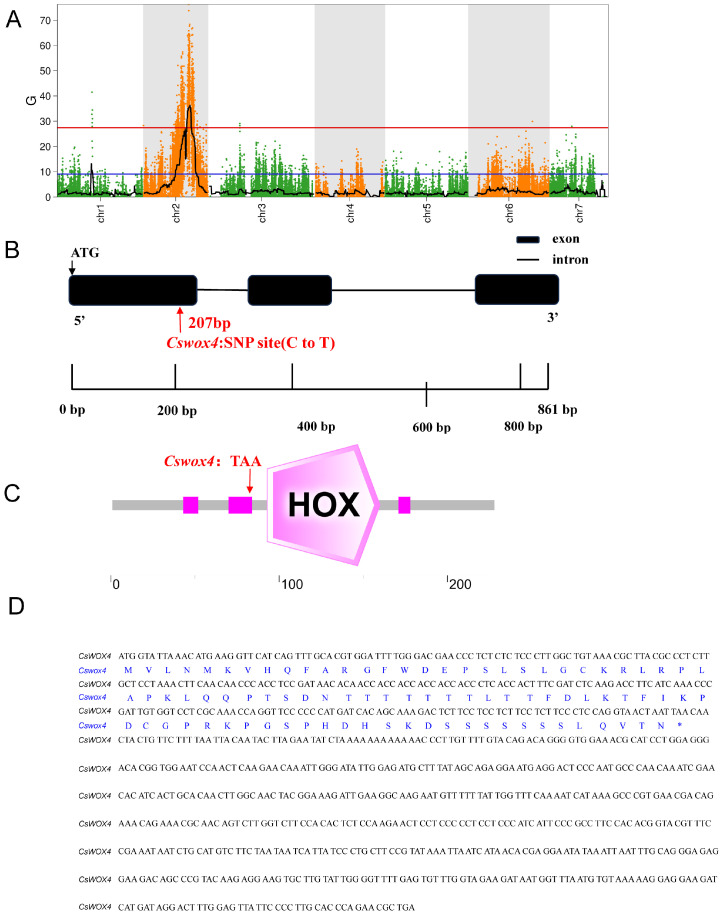
MutMap analysis and gene structure analysis of *CsWOX4*. (**A**) The distribution of the G-index in 7 chromosomes. The horizontal axis represents the name and length of each chromosome, the vertical axis represents the G-index value, and the red line represents the threshold line corresponding to 95%. (**B**) Gene structure of *CsWOX4*. Black boxes represent exons and black lines represent introns. (**C**) Predicted protein domain of *CsWOX4*. (**D**) Coding sequence and amino acid sequence alignment of *CsWOX4*. Black represents the complete sequence information of *CsWOX4,* blue represents the amino acids expressed by the mutant cswox4, and * represents the stop codon of *cswox4*.

BSA was used to map the mlt locus in a framework. The Δ SNP index method was used to calculate significant associated sites, and the associated region was located within the range of 16,140,001–1,888,000 on chromosome 2, containing information regarding 5711 genes (Figure 2A). Subsequently, gene annotation and secondary result prediction analyses were performed based on the differential gene expression determined using parental transcriptome sequencing. By intersecting these results with the aforementioned gene regions, through annotation analysis, sequence cloning and the expression level detection of some candidate genes, the candidate gene *CsWOX4* was ultimately identified within this specific range of genes.

### 2.3. Cloning and Sequence Analysis of CsWOX4

The bioinformatics analysis revealed that CsWOX4 mainly functions as a transcription factor, and mutated Cswox4 may still possess a transcription factor function. The cucumber CsaV3_2G026510 gene was named *CsWOX4* because the derived protein sequence shared the highest similarity with *AtWOX4* in *Arabidopsis*. The cDNA sequences of the candidate genes cloned from K39 and J128 were encoded, and the amino acid sequences of J128 and K39 were derived. The DNA sequence of CsaV3_2G026510 in J128 spans 2161 bp and consists of three exons and four introns (Figure 2B). The encoded protein consists of 285 amino acid residues and contains the structure of the HOX gene family (Figure 2C). The sequence of the K39 mutant showed a base mutation after position 264, with a TAA mutation in TGA leading to the premature termination of its sequence and encoding only 88 amino acid sequences, which severely affected the gene function and conserved domains. After comparing the amino acid sequences of the proteins encoded by the *CsWOX4* and *Cswox4* genes, *Cswox4* was found to be truncated and lacked 113 amino acids, including the conserved WUS box sequence, but still retained a typical HD-type transcription factor conserved domain. Notably, the morphology of the plant changes significantly following mutation in *CsWOX4* (Figure 2D). Therefore, we studied the differences in the *CsWOX4* and *Cswox4* genes between WT and mutant plants to investigate the causes of the mutations.

### 2.4. Subcellular Localization of CsWOX4 Protein

**Figure 3:**
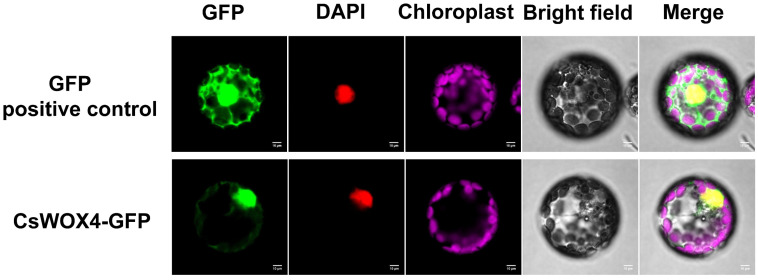
Subcellular localization of *CsWOX4* protein. Green fluorescent protein GFP: excitation light 488 nm, emission light 510 nm. Red fluorescent protein mKATE excitation light 561 nm emission light 580 nm. Scale bar 10 μm.

To verify the expression site of CsWOX4, the Golden Gate method was used to construct a *CsWOX4* and GFP protein fusion expression vector. The protoplasts of *N. benthamiana* seedlings were extracted, and the CsWOX4–GFP protein fusion expression vector plasmid, nuclear marker and cytoplasmic marker were transformed into tobacco protoplasts. Fluorescence confocal microscopy demonstrated that the green fluorescence signal of GFP completely overlapped with the red fluorescence of the nuclear marker, indicating that the CsWOX4–GFP protein was localized in the nucleus. In addition, weak green fluorescence was observed in the cytoplasm, indicating that CsWOX4–GFP was also expressed in small amounts in the cytoplasm (Figure 3).

### 2.5. Expression Pattern of CsWOX4

**Figure 4:**
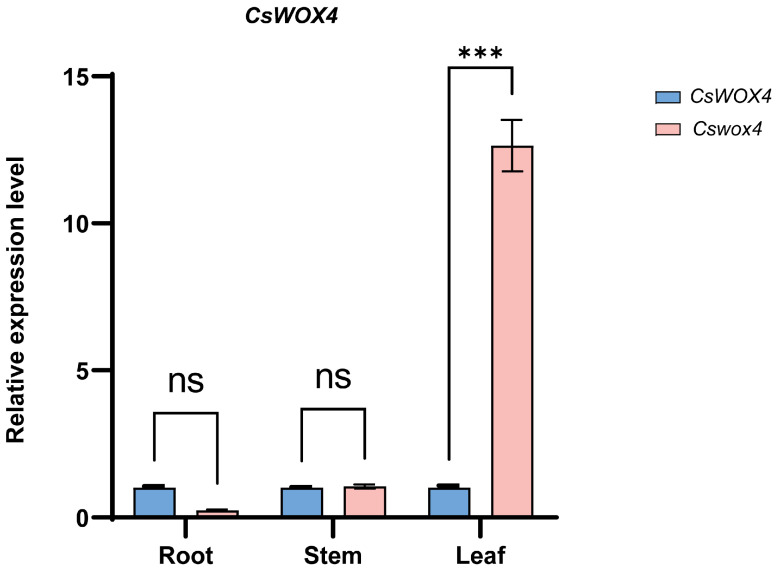
Expression profiles of *CsWOX4* gene in different tissue types of J128 and K39 mutants detected by qPCR. Values shown are mean ± SD calculated from three biological and three technical replicates. Statistical significance is denoted as *** for *p* < 0.001 as determined by Student’s *t*-test.

*CsWOX4* was expressed in both the WT and mutant plants, with the highest expression observed in the leaves of the WT plants. Subsequently, we compared the *CsWOX4* expression in different organs between WT J128 and mutant K39 plants. Compared with WT J128, the *CsWOX4* expression in the leaves of mutant J128 was significantly reduced, with 12.5 times higher expression in WT leaves than mutant leaves. Furthermore, the *CsWOX4* expression was slightly higher in the roots of the WT (7.5 times) than in the roots of the mutant (Figure 4). No significant differences in expression were observed in the stems of the WT and mutant plants. Furthermore, similarly to *CsWOX4*, *Cswox4* was expressed in the roots, stems and leaves, with the highest expression in the leaves and almost no expression in the roots. The *Cswox4* expression in the mutant was significantly lower than that of *CsWOX4* in the WT. The amino acid sequence alignment analysis revealed that Cswox4 had a short amino acid sequence and the conserved deletion after the truncation of the WUS box structural domain. The WUS box primarily functions as a transcriptional repression domain for the regulation of stem apex stem cells. The stability of cell populations plays an important role in floral organ development. The site of action of the *Cswox4* gene did not change after mutation, which may have been due to the deletion of the conserved sequence in the WUS box, resulting in the lack of suppression of the *Cswox4* expression in the mutant. The appearance of plant phenotypes may be attributed to a sharp increase in *Cswox4* expression, indicating that conserved WUS box sequences play an important role in plant development and *CsWOX4* gene function. However, the emergence of mutants is unclear. Further research is needed to determine whether the emergence of mutants is associated with the increased *Cswox4* expression at the transcriptional level or changes in protein structure caused by the deletion of conserved WUS box sequences.

### 2.6. Protein Expression Detection of CsWOX4

**Figure 5:**
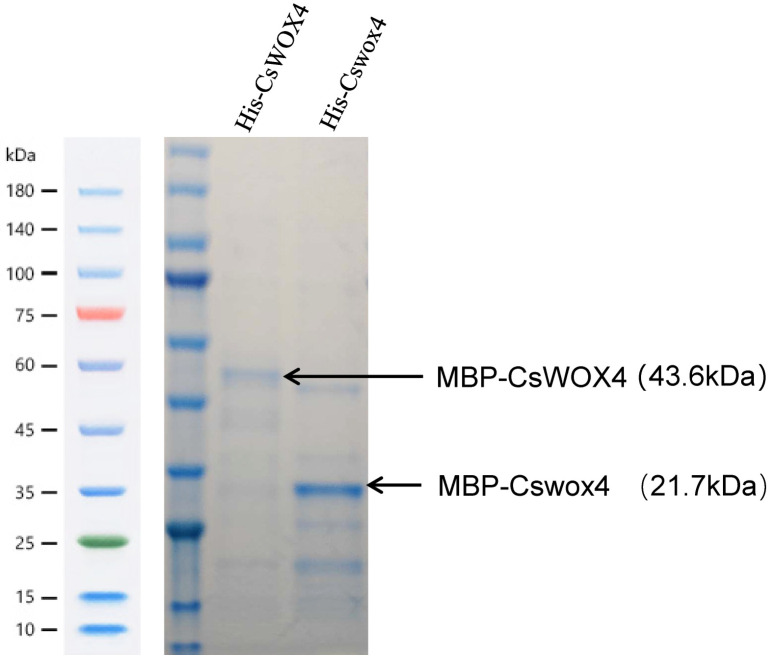
SDS–PAGE analysis of CsWOX4 and Cswox4 fusion proteins. M: On the left side is Protein pre-staining marker; IPTG-induced CsWOX4 fusion protein; IPTG-induced Cswox4 fusion protein.

An expression analysis was performed on small amounts of protein obtained using 10% SDS–PAGE gel. After electrophoresis, the gel was removed and staining was performed using Coomassie brilliant blue, following the manufacturer’s instructions. Subsequently, imaging under white light was performed using a chemiluminescence imaging system (Bio-Rad Laboratories). Specific bands were observed in both the first and second gel holes, as shown in Figure 5. The size of the CsWOX4 protein after wild-type purification was 43.6 kDa, and the size of the Cswox4 protein after mutant purification was 21.7 kDa (Figure 5). The fusion of the GST tags indicated that the encoded protein exhibited a short segment following the *CsWOX4* mutation.

### 2.7. Transcriptome Analysis Revealed the Involvement of CsWOX4 in Regulating Leaf Shape Pathways

**Figure 6:**
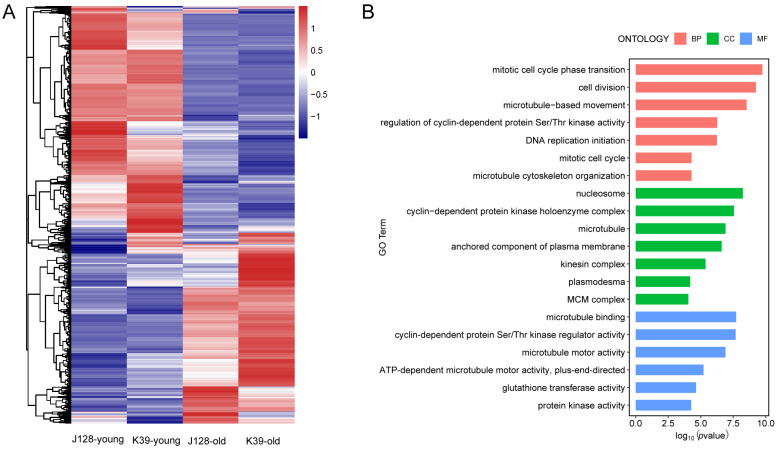
Transcriptome data analysis of J128 and K39. (**A**) Differential expression display of “J128” and K39 genomics. (**B**) Differential gene GO annotation analysis.

The RNA-seq transcriptome analysis of the young and mature leaves of the J128 and K39 mutants revealed 2703 differentially expressed genes (Figure 6A). The GO enrichment analysis performed on the differentially expressed genes revealed that they were mainly enriched in biological processes, such as mitochondrial cell cycle phase transition, cell division, microtubule-based movement, the regulation of cyclin-dependent protein serine and DNA replication initiation; cellular components, such as nucleosomes, cyclin-dependent protein kinase holoenzyme complexes, microtubules and anchored components of plasma membranes; and molecular functions, such as microtubule binding, cyclin-dependent protein serine, microtubule motor activity and ATP-dependent microtubule motor activity. The differential gene clustering exhibited by the molecular functional groups was mainly related to glutathione transferase activity and protein-related enzyme activity processes. Therefore, through GO annotation analysis, the most significant enrichment in biological processes (BP), cellular components (CC) and molecular functions (CF) was found for the mitochondrial cell cycle phase transition, nucleosome and microtubule binding pathways (Figure 6B). Differentially expressed genes due to the CsWOX4 mutation may participate in plant developmental processes in the form of transcriptional regulation, thereby affecting the leaf morphology. The GO annotations were also enriched in many signaling pathways, thus affecting pathway conversion activity, oxidoreductase activity (GO:0016491), transferase activity (GO:0016740), hydrolase activity (GO:0016787) and ion binding (GO:0043167) (Supplementary Figure S1). Multiple indicators revealed that the mutation likely impacted glutathione oxidoreductase, mannan synthase activity and the aspartate-type endoplasmic reticulum.

### 2.8. Analysis of Genes Involved in Leaf Type Regulation

**Figure 7:**
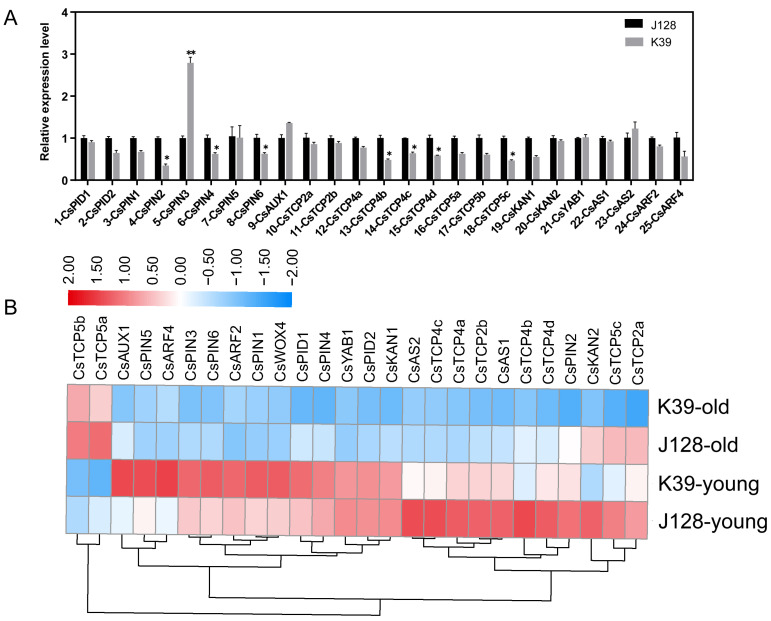
Analysis of key gene expression levels in different regulatory pathways of leaf types. (**A**) qPCR expression level detection of key genes. (**B**) Heat map analysis of key gene expression patterns in transcriptome. Values shown are mean ± SD calculated from three biological and three technical replicates. Statistical significance is denoted as * for *p* < 0.05 and ** for *p* < 0.01, as determined by Student’s *t*-test.

Auxins regulate leaf development by affecting leaf initiation, spatial polarity formation, morphogenesis and edge development. We identified 25 auxin-related regulatory genes in this study (Figure 7A) (Supplementary Table S1). PID is involved in vascular system development in *Arabidopsis* leaves, lateral root formation and the phototaxis response. Therefore, we assessed the expression of *CsPID1* and *CsPID2* in mutant and WT plants and found that the expression of both genes was reduced to varying degrees in the mutant K39. The PIN family is the most extensively studied efflux carrier family in plants. Therefore, we analyzed the expression of *CsPIN1*, *CsPIN2*, *CsPIN3*, *CsPIN4, CsPIN5* and *CsPIN6*. The expression of these genes in J128 generally showed a decreasing trend, although a significant increase in *CsPIN3* expression (2.5 times) was observed relative to that in the WT J128. Therefore, *CsWOX4* may cause changes in the leaf type by regulating *CsPIN3* expression. Functional research on the TCP family has mainly focused on lateral branch development, leaf formation and flower symmetry in plants. Therefore, we analyzed the expression of *CsTCP2a* (Csa6G524000), *CsTCP2b*, *CsTCP4a*, *CsTCP4b*, *CsTCP4c*, *CsTCP4d*, *CsTCP5a*, *CsTCP5b* and *CsTCP5c*. A decreasing trend in the overall TCP family expression was observed in the mutants. The YAB, KAN, ARF and HD-ZIP III genes in *Arabidopsis* directly participate in the development of the near and far axes of leaves. Therefore, we analyzed the expression of *CsAUX1*, *CsKAN1*, *CsKAN2*, *CsYAB1* (Csa5G160210), *CsAS1* and *CsAS2.* Notably, *CsARF2* and *CsARF4* led to a slight increase in *CsAUX1* and *CsAS2* expression, although the expression of the other genes showed a downward trend (Supplementary Table S2). Overall, *CsPIN3* may be a key target for future research on leaf-regulatory mechanisms (Figure 7B).

## 3. Discussion

In the present study, we cloned the regulatory genes of the cucumber leaf type mutant K39 using a map cloning strategy. Our findings revealed that CsaV3_2G026510 is a candidate gene for leaf type regulation. Fine mapping successfully narrowed down the leaf type mutant candidate gene to a 2.8 Mb region (Figure 2). We then conducted preliminary screening through the annotation information and differences in expression and ultimately cloned the CsWOX4 sequence. Notably, only one nonsynonymous mutation (C to T) was identified in the first exon of *CsWOX4* between the mutant and its WT in this region, resulting in an amino acid change that caused the premature termination of translation (Figure 2). The bioinformatics analysis of the *CsWOX4* gene revealed that the Cswox4 shortcut deleted the conserved WUS box sequence, and its expression was significantly upregulated in the mutant. Specifically, the *CsWOX4* gene mutation resulted in a significant change in the morphology of the mutant plants owing to the deletion of the conserved WUS box sequence, and the expression of *Cswox4* was sharply upregulated. Therefore, the appearance of the mutant phenotype may have been caused by the deletion of the conserved WUS box sequence, resulting in changes in the gene protein structure and gene structure, or by the upregulation of *Cswox4* expression, resulting in changes in other development-related genes and affecting plant development (Figure 5). Our in vitro protein expression analysis revealed that the mutated sequence exhibited abnormalities in protein expression, with reduced protein expression and failure to express its main structural domain. It has been reported that *OsWOX4* is a key regulatory factor in the early stages of rice leaf development. The downregulation of *OsWOX4* leads to serious defects in leaf development, such as arrested vascular differentiation, partial defects in early cell proliferation required for midrib formation and the inability to maintain cell activity in thin-walled cells. Therefore, *WOX4* may be involved in regulating the leaf morphology in different species.

The WOX transcription factor family plays a transcriptional regulatory role in plant development, such as in the leaves and flowers of crops [17]. To date, the WOX3 and WOX1 subfamilies have been reported to affect leaf development in the WOX family, mainly including PRS/WOX3 from the WOX3 subfamily in *Arabidopsis* and the NS1 and NS2 genes in maize [18]. The overexpression of tomato SlLAM1 rescued these defects in the tobacco lam1 mutant [19]. The leaves of CsWOX1-OE plants are significantly smaller than those of WT plants. Microscopic observations revealed that the volume of epidermal cells under mutant leaves significantly increased compared with that in the WT, which showed significant decreases. CsWOX1 regulates auxin polar transport genes by directly activating CsPIN2. CsPIN2 mainly functions at the distal ends of leaves. It has been reported that *CsWOX1* has an activating effect on the expression of *CsPIN2*. *CsWOX1* is involved in the formation of leaf vein patterns, which in turn affects leaf shape formation. The mutation of the Cswox1 gene is mainly due to the loss of a “G” at position 248 of exon 4. After the loss, the termination codon TAA appears prematurely, causing the protein to terminate prematurely during translation and become short. This evidence indicates that CsWOX1 influences the cucumber leaf morphology through the polar transport process of auxin [20]. In our work, we also found that CsWOX4 was truncated, causing abnormal protein expression. Therefore, we speculate that this is an important gene affecting the leaf shape.

We performed an RNA-seq analysis of the J128 WT and K39 mutant leaves to comprehensively analyze the possible mechanisms of action in mutant plant development from the perspective of gene expression. Notably, 2703 significantly differentially expressed genes were identified between the J128 WT and K39 mutant samples, and GO enrichment analysis revealed their enrichment in metabolic processes, with transcriptional regulation being the most significant. The GO annotation pathways of differentially expressed genes were closely related to hormone regulation, especially auxin, in the cell cycle and transcription processes. Auxins can participate in various processes, such as plant growth and development, cell division and elongation, embryogenesis and apical dominance [21]. Auxin transport pathways in plants are manifested in two ways. One is short-distance transport, which relies on transport proteins such as AUX/LAX, PIN and ABCB/PGP in phloem cells and vascular tissue, and the other is long-distance transportation, which relies on chemical permeation and transpiration in the phloem [22]. The auxin efflux carrier (PIN), auxin influx carrier (AUX/LAX) and ABCB/PGP participate in the polar transport of auxin, which contributes to the differences in the auxin concentration and generation of the maximum auxin concentration in the region [23,24]. The PIN family is the most extensively studied efflux carrier family in plants [25,26], whereas PIN1 is the most extensively studied member of the PIN protein family. PIN1 is localized on the cell membranes of stem apical meristematic tissue, xylem thin-walled cells and vascular bundle cells [27,28]. The PIN2 protein is involved in root growth in response to gravity [29], whereas the PIN3 protein is involved in gravity growth, phototaxis growth and the lateral transport of auxin [30]. PIN4 is involved in auxin transport in the root tip quiescent center [31]. In this study, the RNA-seq results revealed the significant differential expression of *CsPIN3*. PIN5 is responsible for transporting auxin from the cytoplasm to the endoplasmic reticulum lumen [32]. PIN7 regulates root gravity growth [33]. Members of the AUX/LAX family act as carriers of auxin influx and participate in establishing auxin concentration gradients in different tissue types and organs [34]. Research on the impact of PINOID (PID) on plant development shows that it acts as a protein kinase and plays a positive regulatory role in auxin polarity transport by phosphorylating PIN proteins and controlling PIN polarity localization [35]. OsPID regulates the polar transport of auxin during rice development, and its overexpression leads to the delayed formation of adventitious roots, curled stem growth and phototaxis loss in rice [36]. CsPID participates in ovules by regulating the polar transport of auxin [37]. Functional research on the TCP family has primarily focused on lateral branch development, leaf formation and flower symmetry in plants [38].

Moreover, there have been reports identifying mitochondrial defective mutant Narrow Leaf Fine Grain 1 (nlg1) from mutant populations treated with EMS, which exhibits Narrow Leaf Fine Grain. In addition, nlg1 exhibits an abnormal mitochondrial structure and is sensitive to mitochondrial electron transport chain inhibitors [39]. Research has shown that γ TuRC can affect the nucleation and kinetics of MT in the surface cells of mustard leaves. Gamma TuRC nucleates MT at an angle of approximately 40° towards the positive end of existing MT or primarily in the form of antiparallel beams. A small portion of γ TuRC exhibits mobility and can track the end of MT. When γ TuRCs modify the depolymerization MT end, they decrease the depolymerization rate and ultimately affect the formation of the leaf morphology [40]. In our transcriptome analysis, we also found that WOX4 is associated with mitochondrial genes and microtubule binding and nucleosome organization. These will be a key focus of the next stage of our research.

## 4. Materials and Methods

### 4.1. Plant Material Cultivation, Targeted Population Construction and Analysis

Mutant K39 was identified from natural mutations in the field. Natural mutants and wild-type (WT) plants were hybridized in the field to obtain the F1 generation population. The F2 generation was isolated for statistical analysis and sampling for the BSA localization experiments. Segregating populations were developed by crossing K39 with J128 to investigate the genetic inheritance and map-based cloning of the candidate gene. The F2 segregating population of J128 × K39 plants consisted of 257 individuals for molecular biology research, candidate gene localization and cloning. The deviation from the expected 3:1 separation ratio in the F2 population was evaluated using χ^2^ tests. All cucumber plants were grown under natural conditions in the greenhouse of the Wuqing Innovation Base, Tianjin Academy of Agricultural Sciences, China.

### 4.2. Genetic Analysis and Allelic Test

The F1 generation was obtained by crossing the WT “J128” and mutant “K39”, whereas the F2 population was obtained by self-crossing the F1 generation. The number and separation of WT and mutant leaves were analyzed using chi-squared tests to determine the proportion of the F2 population (χ^2^). Backcross (BC) population BC1 was hybridized with its parents in the F1 generation, resulting in two backcross populations: BC1 (F1 × P1) and BC1 (F1 × P2). The F1 (K39 × J128) population exhibited WT behavior. Cross-validation was performed to obtain subsequent population and phenotypic data using the chi-squared test. The online SMART software (http://smart.embl-heidelberg.de/, accessed on 15 October 2024) was used to predict the protein structures of the mutated genes. The amino acid sequences of the other mutated genes were searched to identify the species and accession numbers in GenBank using NCBI BLAST.

### 4.3. Fine Mapping of mlt

Using the resequencing method, two mixed pools, F2 (J128) and F2 (K39), were sequenced at a depth of 10×. The cucumber genome was used as the reference genome, and 123,170 single-nucleotide polymorphisms (SNPs) were identified between the two mixed pools. Preliminary gene mapping was performed on two genotypic pools using BSA by pooling equal amounts of DNA from 50 WT (J128) and 54 mutant (K39) plants selected from the K39 × J128 F2 population. Sequence alignment was performed using DNAMAN v10.0. Primer Premier v5.0 was used for primer design. Candidate genes were screened based on genome annotation and expression.

### 4.4. Transcriptome Data Analysis

Transcriptome sequencing was performed using HiSeq X Ten on an Illumina sequencing platform. Overexpression, mutant strains and receptor materials were selected to inoculate leaf tissue at different stages to prepare RNA-seq samples. Standard extraction methods were used to extract RNA from the tissue, with stringent measures taken to ensure the quality and integrity of the RNA samples. A NanoPhotometer spectrophotometer was used to detect the RNA purity, and then an Agilent 2100 bioanalyzer was used to accurately detect the RNA integrity (RIN value) and perform the quality control of the raw data. After completing library inspection, different libraries were pooled according to the effective concentration and target data volume requirements for Illumina sequencing. High-quality control data were compared, quantified, analyzed for significant differences and functionally enriched. K-means, WGCNA and other tools were employed to perform co-expression network analyses of genes. Gene Ontology (GO) and Kyoto Encyclopedia of Genes and Genomes (KEGG) functional enrichment analyses of the differentially expressed genes were performed using the GO and KEGG databases, respectively, which identified *CsWOX4* co-expressed genes.

### 4.5. Identification and Gene Cloning of mlt

Genomic DNA was extracted from the WT J128 and K39 mutants using the TaKaRa (Beijing, China) MiniBEST Plant Genomic DNA Extraction Kit (9768S) to identify candidate genes for the cloning and regulation of leaf types. Preliminary analysis was conducted on the full-length genome sequence of CsaV3_2G026510 in cucumber variety 9930 based on genome annotation information. Primers were designed to amplify the J128 and K39 sequences. Total RNA was extracted from the roots, stems and leaves of the WT J128 and K39 mutants. An RNA extraction kit (TaKaRa) and firststrand were used to synthesize cDNA using reverse transcription (PrimeScript). An RT reagent kit (RR037Q, Perfect Real Time, Beijing, China) was used to clone and encode the candidate gene sequences. PCR amplification was performed with an iCycler thermal cycler (Bio-Rad Laboratories, Hercules, CA, USA) using a PrimeStar HS DNA Polymerase Kit (R045Q, Takara Bio, Beijing, China)). Subsequently, a gel extraction kit (9762, TaKaRa MiniBEST Agarose Gel DNA Extraction Kit Ver.4.0 (Beijing, China)) was then used to extract the bands. Next, sequencing was performed using Genewiz (Suzhou, China), and the sequences were compared with Snapgene. All primers were designed using Primer Premier v5.0, and the primer sequences are listed in Supplementary Table S2. PCR conditions: 94 °C, 5 min→(94 °C, 30 s→55 °C, 45 s→72 °C, 30 s) × 30 cycles→72 °C, 10 min→4 °C. 

### 4.6. Subcellular Localization

*Nicotiana benthamiana* seedlings were cultivated at approximately 25 °C for 15–20 days. Several leaves were mixed with 5–10 mL of enzymatic solution and soaked. Enzymatic hydrolysis was performed at 24 °C for 4 h. The sample was centrifuged at 300 rpm for 3 min to remove the supernatant, washed twice with 10 mL of precooled W5 solution and centrifuged at 4–25 °C. An appropriate amount of MMG solution (0.5–2 mL) was added as needed for the suspension (100 μL of protoplasts was added separately, for a total of 200 μL of protoplasts) until the concentration reached 2 × 10^5^ cells/mL. Microscopic examination revealed that the protoplasts were round and showed slight rupturing. Then, the protoplast suspension was added to 10 μL of DNA. If co-transformed, 200 μL of protoplast suspension was added to 20 μL of DNA (10 μL of target gene vector plasmid and 10 μL of marker plasmid). An amount of PEG4000 solution (110 μL) equal to the sum of the DNA and protoplast volume was mixed gently and evenly and allowed to stand at room temperature for 10–15 min. The reaction was terminated by adding 1 mL of W5. The protoplasts were collected, and the supernatant was removed. Then, W5 was added to the solution, which was washed 1–2 times. Finally, the W5 solution was added and incubated in the dark at 25 °C for 18–24 h. The supernatant was then removed, and approximately 100 μL of the protoplast solution was used for fluorescence and laser confocal microscopy.

### 4.7. Prokaryotic Expression and Analysis of CsWOX4 and Cswox4

Protein purification was performed using 30 µL of the sample, which was added to 10 µL of SDS sample buffer (4×) and mixed well. The solution was boiled at 100 °C for 10 min, the sample was loaded, and the preliminary SDS–PAGE analysis of the induced expression results was performed. The expression induction analysis revealed that the target protein His-Cswox4 was significantly expressed in the supernatant at 18 °C. The preserved bacterial solution was activated and inoculated into 400 mL of LB medium until the OD600 reached approximately 0.6. Then, 0.2 mM IPTG was added, and expression was induced at 18 °C for 16 h. Bacterial cells were collected from the center. A lysis buffer (1 g of bacterial cells added to 5 mL of lysis buffer) was used to resuspend the bacterial cells for ultrasonic fragmentation. Cell lysates were collected, filtered and then incubated with the corresponding beads at 4 °C for 3 h. The beads were then collected (supernatant could be stored first), and a wash buffer was added for washing. If the Bradford detection solution changed color and appeared blue, the washing frequency was increased accordingly until no color change was observed. Finally, gradient elution was performed using an elution buffer (imidazole), and the eluent was collected. Next, 30 µL of eluent was added to 10 µL SDS sample buffer (4×) and boiled for 10 min. The samples were then loaded and analyzed using SDS–PAGE with the following settings: concentrated gel at 80 V for 30 min, separation gel at 120 V for 60 min, the amount of the protein loaded/lane was 40 µL and the electrophoresis time was adjusted appropriately. Thereafter, the gel was removed from the glass plate, and the concentrated glue was removed and placed into the dyeing solution for incubation at room temperature for 10 min. The staining solution was then removed, and the samples were washed using tap water at room temperature for 30 min and photographed.

### 4.8. Gene Expression Analysis Using q-PCR

RNA was extracted using a plant RNA extraction kit (TaKaRa MiniBEST Plant RNA Extraction Kit (9769S Takara, Beijing, China)), following the manufacturer’s instructions, and stored at −80 °C. cDNA was prepared using A Plus All In One 1st Strand cDNA Synthesis SuperMix (gDNA Purge), following the manufacturer’s instructions. Briefly, 20 μL of the reaction mixture was prepared on ice, and further steps involved incubation at 50 °C for 15 min and then at 85 °C for 5 s and cooling on ice. The obtained cDNA was stored at −20 °C until further use. The experimental data were normalized using the WT as the control group. Pre-denaturation was performed at 94 °C for 30 s in 1 cycle, followed by denaturation at 94 °C for 5 s, annealing at 58 °C for 15 s and extension at 72 °C, with 40 cycles. After 40 cycles, the specificity of amplification was verified by melting curves (65 °C to 95 °C, increments of 0.5 °C). The calculation formulas used were as follows: A = CT (target gene, test sample) − CT (reference gene, test sample), B = CT (target gene, control sample) − CT (reference gene, control sample) and ΔΔCT = A − B relative expression = 2^−ΔΔCT^.

## 5. Conclusions

In summary, we determined the mechanism through which *CsWOX4* regulates the cucumber leaf morphology The co-upregulation of *CsWOX4* and *CsPIN3* expression may regulate the production of the cucumber maple leaf type by participating in auxin polar transport. CsWOX4 can participate in the development of the distal regions of leaves together with the expression of CsPIN family members, TCP family members, CsPID and other genes, forming a feedback regulatory mechanism to regulate leaf cell development. In this study, we identified the leaf type regulatory gene *CsWOX4* and conducted a preliminary analysis of its expression pattern, exploring its possible regulatory pathways. This work is meaningful in understanding the growth and development of cucumber. Our results indicate that CsWOX4 is upregulated in the roots. The next step of our work plan is to explore the tissue-specific regulatory mechanisms and the possible regulatory network of CsWOX4 as a whole.

## Figures and Tables

**Table 1:** Genetic analysis of mutant phenotype trait in F1 and F2 populations.

Generation	Total	Mutant Type	WT	Segregation Ratio	Chi-Squared Value
P1 (J128)	100	0	100		
P2 (K39)	108	108	0		
F1 (P1 × P2)	70	70	0		
F1 (P2 × P1)	0	0	0		
BC1 (F1 × “J128”)	214	0	214		
BC1 (F1 × “K39”)	234	115	119	0.96:1	
F2	257	66	191	1:2.894	0.03

## Data Availability

This study has provided all generated and analyzed data as requested. The sequence data of MutMap have been deposited in the NCBI Sequence Read Archive (SRA) repository under accession number PRJNA1173244. The RNA-seq data are stored in the NCBI Sequence Read Archive (SRA) repository under accession number PRJNA1171965.

## Supplementary Materials

The following supporting information can be downloaded at: https://www.mdpi.com/article/10.3390/ijms252212189/s1.
