# Supplementary material for: Functional Analysis of CsWOX4 Gene Mutation Leading to Maple Leaf Type in Cucumber (Cucumis sativus L.)
Source: Int J Mol Sci. 2024 Nov 13;25(22):12189. doi: 10.3390/ijms252212189 (PMC11595286; doi:10.3390/ijms252212189)
Supplement: Supplementary file 1 [file ijms-25-12189-s001.zip › ijms-3288487-supplementary.pdf]

Supplymental Figure S1. Go annotation Pathway analysis.

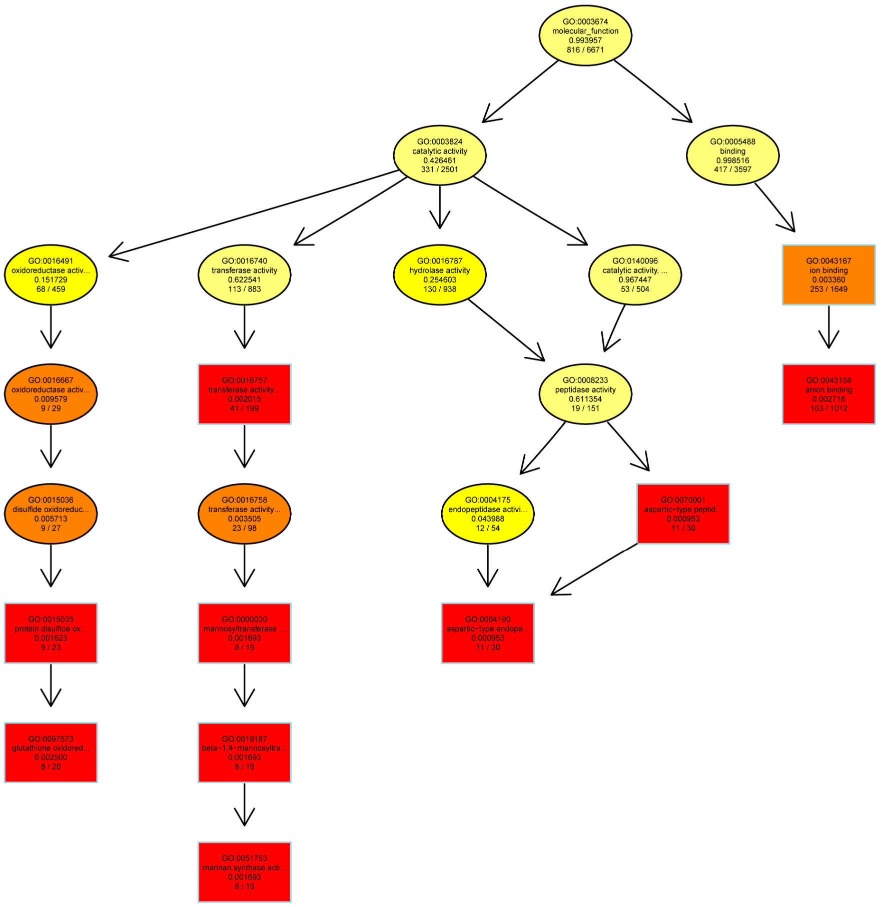


Supplementary Table S1. Gene description

| Gene Name | Gene Symbol |
|---|---|
| CsWOX4 | CsaV3_2G026510 |
| CsPID1 | Csa1G537400 |
| CsPID2 | Csa2G006100 |
| CsPIN1 | Csa1G042820 |
| CsPIN2 | Csa5G284520 |
| CsPIN3 | Csa1G025070 |
| CsPIN4 | Csa4G430820 |
| CsPIN5 | Csa1G427480 |
| CsPIN6 | Csa5G576590 |
| CsAUX1 | Csa4G308640 |
| CsTCP2a | Csa6G524000 |
| CsTCP2b | Csa1G025920 |
| CsTCP4a | Csa4G088720 |
| CsTCP4b | Csa6G156050 |
| CsTCP4c | Csa4G628330 |
| CsTCP4d | Csa1G077150 |

| | |
|---|---|
| CsTCP5a | Csa4G132680 |
| CsTCP5b | Csa1G014310 |
| CsTCP5c | Csa1G039270 |
| CsKAN1 | Csa3G194380 |
| CsKAN2 | Csa3G008330 |
| CsYAB1 | Csa5G160210 |
| CsAS1 | Csa3G734210 |
| CsAS2 | Csa2G070920 |
| CsARF2 | Csa1G000580 |
| CsARF4 | Csa6G291920 |

Supplementary Table S2. Primer Sequence

| Primer Name | Forward Primer (5′→ 3′) | Reverse Primer (5′→ 3′) |
|---|---|---|
| Actin | ACTGTGCTGTCCTCATTATTG | AGGGTGAAAGCAAGAAGAGC |
| CsWOX4-CDS | CCAAACACCACCTCATTTCCCACTT | CATTATCTTCTACCAAACACTCAAA |
| CsWOX4-104 | CCCAACAAATCGAACACATCAC | CTGTCGTTCACGGGCTTTAT |
| 1-CsPID1-86 | AGAGACCTTAAACCGGAGAATG | GCGTCGGAACAGAGAGATAAA |
| 2-CsPID2-109 | CATGCTCTCCGATTTCGATCT | GAAGCTGTAGGAGGAGGATCTA |
| 3-CsPIN1-94 | GGTCTCACTTGGTCCTTAGTTT | CAAGCCCTGCATCAGATAGT |
| 4-CsPIN2-108 | GGGAGTTTGGAGTCGATGATTA | GCTTCCAGAGTTACCTCCATAC |
| 5-CsPIN3-105 | CAGGACAGTACAAGATGGATGAG | GGCTTCCAAACACATCAGAAAC |
| 6-CsPIN4-92 | GTTGATTGCAGAGCAGTTTCC | ACGGCTCTTTCCCATCTAAAG |
| 7-CsPIN5-95 | CGACGAGGAGATGTTGAAGAAG | GGTTTGGTGGTGGGTAAGAT |
| 8-CsPIN6-94 | GAGACTGATGCTGAGATTGGAG | GAAGTGAACAAGGTCCAAGAGA |
| 9-CsAUX1-120 | CCACTGTCTTCATCCCTTCTTT | CTTCAGTCTGGCCGTGAATAA |
| 10-CsTCP2a-102 | GAGCAGAAGCAAAGCCAATTAC | CTCAGATCGCGAAAGAGACAA |
| 11-CsTCP2b-116 | ACTGCGAAAGAGAAGGAAAGAG | GTGTTATTGGCGCCGTTATTG |
| 12-CsTCP4a-99 | GTTCCCGCGGATAACTTCTT | TGCGGCGAGTTGAATACATAG |
| 13-CsTCP4b-124 | AATCTACGGTGCCGCATTAC | GGCAACTCGTTGGAGATGAA |
| 14-CsTCP4c-107 | TCTCTGGAACAACACCATTAGG | CACTGTGCTCCATTGCAATATC |
| 15-CsTCP4d-101 | AAAGGCAACCTCTCTGTCTTC | GCTCCGATATTGCTCTGACTT |
| 16-CsTCP5a-114 | CGGCCTTCACTACCAATCAA | TGTTTCTGTTCGGTCTCATCC |
| 17-CsTCP5b-104 | CTCTCCCTTCCACCAACAAA | GAGAAGAGGAAGAAGAGGAAGAAG |
| 18-CsTCP5c-91 | CAAGAACGTGTTCAAGACAAGG | CTGTGCCTATCTTTACCTCCAA |

| | | |
|---|---|---|
| 19-CsKAN1-138 | CTTCTTCGTCGTCCTCTTCTTC | CGGTTTGAGTACGAGAGTCATT |
| 20-CsKAN2-118 | GCGGTGGTAGTCAGTTTAGTT | AAACGAGCATGGAGAGTAGTG |
| 21-CsYAB1-134 | CATCAGCTTGGCCATTCTTTC | GTAACTCCTCGAGATGGCATAC |
| 22-CsAS1-104F | CATGTGGTGGTAGCCTCTTT | CCTCCATGCTGTTTCCTTCT |
| 23-CsAS2-103 | TCCCTCGAGATCAACAACAAC | CCGATACTTGCGGTGACATTA |
| 24-CsARF2-99 | CGGGCTGGTGACTTATCTTATC | ATGAGGCTGCTCAGTTCTATG |
| 25-CsARF4-92 | CTTAACGGTCCGATGGGATAAA | CACTCAAAGGCGGAAGAGAA |
